# Learned Practical Guidelines for Evaluating Conditional Entropy and Mutual Information in Discovering Major Factors of Response-vs.-Covariate Dynamics

**DOI:** 10.3390/e24101382

**Published:** 2022-09-28

**Authors:** Ting-Li Chen, Hsieh Fushing, Elizabeth P. Chou

**Affiliations:** 1 Institute of Statistical Science, Academia Sinica, Taipei 11529, Taiwan; 2 Department of Statistics, University of California, Davis, CA 95616, USA; 3 Department of Statistics, National Chengchi University, Taipei 11605, Taiwan

**Keywords:** Categorical Exploratory Data Analysis, curse of dimensionality, Hierarchical clustering, interacting effects, K-means, LASSO

## Abstract

We reformulate and reframe a series of increasingly complex parametric statistical topics into a framework of response-vs.-covariate (Re-Co) dynamics that is described without any explicit functional structures. Then we resolve these topics’ data analysis tasks by discovering major factors underlying such Re-Co dynamics by only making use of data’s categorical nature. The major factor selection protocol at the heart of Categorical Exploratory Data Analysis (CEDA) paradigm is illustrated and carried out by employing Shannon’s conditional entropy (CE) and mutual information (I[Re;Co]) as the two key Information Theoretical measurements. Through the process of evaluating these two entropy-based measurements and resolving statistical tasks, we acquire several computational guidelines for carrying out the major factor selection protocol in a do-and-learn fashion. Specifically, practical guidelines are established for evaluating CE and I[Re;Co] in accordance with the criterion called [C1:confirmable]. Following the [C1:confirmable] criterion, we make no attempts on acquiring consistent estimations of these theoretical information measurements. All evaluations are carried out on a contingency table platform, upon which the practical guidelines also provide ways of lessening the effects of the curse of dimensionality. We explicitly carry out six examples of Re-Co dynamics, within each of which, several widely extended scenarios are also explored and discussed.

## 1. Introduction

The majority of scientific fields, such as biology [1], neuroscience [2], medicine, sociology and psychology [3] and many others [4], involve dynamics of complex systems [5,6]. Scientists and experts in such fields typically can only imagine or even briefly outline various potential response-vs.-covariate (Re-Co) relationships in an attempt to characterize the dynamics of their complex systems of interest [7]. Given that no explicit functional form of such Re-Co relationships is available, such scientists still go ahead and collect structured data sets by investing great efforts in choosing which features for the role of response variable, and which features for the role of covariate variables. Such choices of features are indeed critical for the sciences because their successes rely entirely on whether such structured data sets can embrace the essence of the targeted Re-Co dynamics or not.

After scientists achieve their scientific quests by generating structured data sets upon the complex systems of interest, it becomes not only very natural, but also very important to ask the following specific question: When such structured data sets are in the data analysts’ hands, what is the most essential common goal of data analysis? This goal is certainly not aimed at an explicit system of equations, nor at a complete set of functional descriptions of the targeted Re-Co dynamic. Instead, this goal can and shall be oriented to decode the scientists’ authentic knowledge and intelligence about the complex systems of interest, and one step further to go beyond the current state of understanding.

In sharp contrast, nearly all statistical model-based data analyses on any structured data sets pertaining to wide-range of Re-Co dynamics always assume an explicit functional structure linking the response variables to covariate variables, including hypothesis testing [8], analysis of variance (ANOVA) and the many variants of regression analysis [9,10], including generalized linear models and log-linear models [11,12]. By framing rather complex Re-Co dynamics with rather simplistic explicit functional structures, statistical model-based data analysis surely will run the dangers of hijacking data’s authentic information content. With such dangers in mind, it is natural to ask the reverse question: What if we can reformulate all fundamental statistical tasks to fit under a framework of response-vs.-covariate (Re-Co) dynamics without explicit functional forms and extract data’s authentic information content of data sets?

As the theme of this paper, we demonstrate a positive answer to the above fundamental question. The chief merits of such demonstrations are that we not only can do nearly all data analysis without statistical modeling, but more importantly we can reveal data’s authentic information content to foster true understanding about the complex systems of interest. Our computational developments are illustrated through a series of 6 well-known statistical topic issues with increasing complexity. All successfully revealed information content is visible and interpretable.

The positive answer resides in the paradigm called Categorical Exploratory Data Analysis (CEDA) with its heart anchored at a major factor selection protocol, which has been under developing in a series of published works [13,14,15,16] and a recently completed work [17]. For demonstrating the positive answer, this paper establishes practical guidelines for evaluating Theoretical Information Measurements, in particular Shannon’s conditional entropy (CE) and mutual information between the response variables and covariate variables, denoted as I[Re;Co] [18], which are the basis of CEDA and major factor selection protocol.

Along the process of establishing such computational guidelines, we characterize four theme-components in CEDA and the major factor selection protocol:

TC-1. Our practical guidelines are established here for evaluating CE and I[Re;Co] without requiring consistent estimations of their theoretical population-version of measurements.
TC-2. All entropy-related evaluations are carried out on a contingency table platform, so learned practical guidelines also provide ways of relieving from the effects of the curse of dimensionality and ascertaining for [C1:confirmable] criterion, which is a kind of relative-reliability.
TC-3. CEDA is free of man-made assumption and structures, so consequently its inferences are carried out with natural reliability.
TC-4. CEDA only employs data’s categorical nature, so the confirmed collection of major factors indeed reveals data’s authentic information content disregarding data types.

The theme-component [TC-1] allows us to avoid many technical and difficult issues encountered in estimating the theoretical information measurement [19,20]. [TC-1] and [TC-2] together make CEDA’s major factor selection protocol very distinct to model-based feature selection based on mutual information evaluations [21,22,23,24], while [TC-3] makes CEDA’s inferences realistic, and [TC-4] makes CEDA to provide authentic information content with very wide applicability.

For specifically illustrating these four theme-components, we consider a structured data set consisting of data points that are measured and collected in a L+KD vector format with respect to L+K features. The first *L* components are the designated response (Re) features’ measurements or categories, denoted as $Y={\left(Y_{1},\ldots ,Y_{L}\right)}^{\prime }$, and the rest of *K* components are *K* covariate (Co) features’ measurements or categories, denoted as $\left\{V_{1},\ldots ,V_{K}\right\}$. It is essential to note that some or even all covariate features could be categorical. Thus, data analysts’ task is prescribed as precisely extracting the authentic associative relations between Y and $\left\{V_{1},\ldots ,V_{K}\right\}$ based on a structured data set.

By extracting authentic associations between response and covariate features, various Theoretical Information Measurements are employed under the structured data setting in [13,14,15,16,17]. In particular, Re-Co directional associations developed in CEDA and its major factor selection protocol rely on evaluations of Shannon conditional entropy (CE) and mutual information (I[Re;Co]) that are all carried out upon the contingency table platform. This platform is indeed very flexible and adaptable to the number of features on row- and column-axes as well as the total size of data points. Such a key characteristic makes CEDA very versatile in applicability. We explain in more detail as follows.

On the response side, a collection of categories of response features (pertaining to Y) is determined with respect to their categorical nature and sample size. Likewise, on the covariate side, a collection of categories for each 1D covariate feature (pertaining to $V_{k}$ for k=1,…K) is chosen accordingly. It is noted that a continuous feature is categorized with respect to its histogram [25]. If L>1, then the entire collection of response categories will consist of all non-empty cells or hypercubes of *L*D contingency tables. However, when *L* is large, the total number of *L*D hypercubes could be too large for a finite data set in the sense that many hypercubes are occupied by very few data points. This is known as the effect of the curse of dimensionality. To avoid such an effect, clustering algorithms, such as Hierarchical clustering or K-means algorithms, can also be performed for fusing the *L* response features (upon their original continuous measurement scales or their contingency tables when involving categorical ones) into one single categorical response variable. The number of categories can be pre-determined for K-means algorithm or determined by cutting a Hierarchical clustering tree in a fashion such as there is only one tree branch per category. The essential idea behind such feature-fusing operations is to retain the structural dependency among these *L* response features, while at the same time reducing the detrimental effect of the curse of dimensionality.

In contrast, singleton and joint (or interacting) effects of all possible subsets of $\left\{V_{1},\ldots ,V_{K}\right\}$ are theoretically potential on the covariate side. However, it is practically known that any high order interacting effects needed to be considered are to a great extent determined by the sample size. That is, a covariate-vs.-response contingency table platform can vary greatly in dimensions: large or small. When viewing a contingency table as a high-dimensional histogram, which is a naive form of density estimation, the curse of dimensionality, or so-called finite sample phenomenon, is supposed to affect our conditional entropy evaluations whenever this table’s dimension is large relative to data’s sample size. We use the notation C[A−vs.−Y] (rows-vs.-columns) for a contingency table of a covariate variable subset $A\subseteq \{V_{1},\ldots ,V_{K}\}$ and response variable Y. As a convention, the categories of Y are arranged along its column-axis, while the categories of *A* are arranged along the row-axis. This row-axis would expand with respect to memberships of *A*.

In CEDA, the associative patterns between any $A\subseteq \{V_{1},\ldots ,V_{K}\}$ and Y would be discovered and evaluated ucing the contingency table C[A−vs.−Y]. It is necessary to reiterate that C[A−vs.−Y] can be viewed as a “joint histogram” or “density estimation” of all features contained in *A* and Y. From this perspective, when the dimension of C[A−vs.−Y] increasingly expands as *A* including more variables, it is expected that its dimensionality would affect the comparability and reliability of conditional entropy evaluations. Consequently, for comparability purposes, this criterion [C1:confirmable] in CEDA arises. This criterion is based on a so-called data mimicking operation developed in [14], as will be described in the following paragraphs.

Let $\tilde{A}$ denote one mimicry of *A* in the ideal sense of having the same deterministic and stochastic structures. In other words, $\tilde{A}$ is generated to have the same empirical categorical distribution of *A*, see [14] for construction details. More practically speaking, if the empirical categorical distribution of *A* is represented by a contingency table, then, given the observed vector of row-sums, $\tilde{A}$ would be another contingency table that has the same lattice dimension and all its row-vectors are generated from Multinomial distribution with parameters specified by the corresponding row-sum and the corresponding vector of observed proportions in *A*’s contingency table. It is noted that $\tilde{A}$ is constructed independent of Y, that is, $\tilde{A}$ is stochastically independent of Y [14].

Denote the mutual information of Y of *A* be I[Y;A] based on C[A−vs.−Y], and likewise $I[Y;\tilde{A}]$ based on $C[\tilde{A}-vs.-Y]$. The [C1:confirmable] used in CEDA is referred to as the degree of certainty that I[Y;A] is far beyond the upper limit of confidence region based on the empirical distribution of $I[Y;\tilde{A}]$. This [C1:confirmable] criterion is indeed in accordance with CEDA’s theme components: [TC-2] and [TC-3], regarding the merits of a contingency table platform in dealing with the curse of dimensionality and facilitating reliability. It is critical to note that we are not estimating the theoretical mutual information of Y and *A* here, and we just want to computationally make sure that I[Y;A] is significantly above zero with great reliability under the reality of having only a finite amount of data points at hand.

Henceforth, it is a critical fact in all applications of CEDA: a covariate feature set is confirmed as having effects on Y only when the [C1: confirmable] criterion of I[Y;A] is established. This concept makes possible for [TC-1] by doing without the nonparametric estimations of Shannon entropy for a continuous distribution function as well as the mutual information for two sets of continuous variables, which have been the long standing problems in physics and neural computing (see theoretical details in [19] and computational protocols based on biGamma function in [20]).

Here, we do not take the view of contingency table as a setup of Grenander’s Method of Sieves (MoS) [26] in this paper. Though MoS can be a choice for practical reasons and computing issues involving many dimensional features or variables, we do not concern primarily on estimating the population-versions of CEs and I[Re;Co] per se, nor the induced sieves biases. Rather, the dimensions of contingency tables are made adaptable to the necessity of accommodating multiple covariate feature-members in *A*. Within such cases, the collection of categories of *A* might be built based on hierarchical or K-means clustering algorithms. From this perspective, computations for theoretical conditional entropy and mutual information between multiple dimensional covariate and possibly multi-dimensional *Y* are neither realistically nor practically possible, due to the limited size of the available data sets. Since this kind of sieves is data dependent, the computations for sieve biases can be much more complicate than that covered in [19].

In this paper, we illustrate and carry out CEDA coupled with its major factor selection protocol through a series of 6 classic statistical topic examples, within each of which various scenarios are also considered. By building contingency tables across various dimensions with respect to different sample sizes, we attempt to reveal the robustness of CEDA resolutions to statistical topic issues. On one hand, we learn practical guidelines of evaluating conditional (Shannon) entropy and mutual information along this illustrative process. On the other hand, we demonstrate that very distinct CEDA resolutions to these classic statistical topic issues can be achieved by coherently extracting data’s authentic information content, which is the intrinsic goal of any proper data analysis. That being said, if modeling is indeed a necessary step within a scientific quest, then data’s authentic information content surely will better serve its purpose by relying on confirmed structures to begin with a new kind of data-driven modeling.

At the end of this section, we briefly project the applicability of our CDA approach for data analysis related to complex systems. One critical application is in a case-control study. Since such studies likely involve multiple features of any data types as often conducted in medical, pharmaceutical, and epidemiological research. Another critical application of CEDA is to serve as an alternative approach to all kinds of regression analysis techniques based on linear, logistic, log-linear, or generalized linear regression models. Such modeling-based analyses are often required and conducted in biological, social, and economic sciences, among many other scientific fields. Furthermore, in our ongoing research, we look into the issue of how well CEDA would deal with causality issues. Addiotonally, with such a wide spectrum of applicability, we project that CEDA will become an essential topic of data analysis education in the fields of statistics, physics, and beyond in the foreseeable future.

## 2. Estimations of Mutual Information between One Categorical and One Quantitative Variables

In this section, we demonstrate how to resolve classic statistical tasks by discovering major factors based on entropy evaluations. First, we frame each classic statistical task into precisely stated Re-Co dynamics. Secondly, we compute and discover major factors underlying this Re-Co dynamics. Inferences are then performed under [C1:confirmable] criterion across a spectrum of contingency tables with varying designed dimensions. Thirdly, we look beyond the setting of the discussed examples to much wider related statistical topics.

Throughout this paper, all 95% confidence ranges (CR) are calculated as the region between 2.5% percentile on the lower tail and 97.5% percentile on the upper tail of any simulated distribution. This CR reflecting both tail behaviors is considered informative. Since even when the upper tail is the only quantity of interest as being the case in this paper, the classic one-sided 97.5% confidence interval becomes visible.

### 2.1. [Example-1]: From 1D Two-Sample Problem to One-Way and Two-Way ANOVA

Consider a data set consisting of quantitative observations $\left\{Y_{lj}|l=1,2;j=1,\ldots ,N_{i}\right\}$ of 1D response feature *Y* derived from two populations labeled by l=1,2, respectively. Let $Y_{lj}$ be distributed according to $F_{l}\left(.\right)$. Testing the distributional equality hypothesis $H_{0}:F_{1}\left(y\right)=F_{2}\left(y\right),\;\forall y\in R^{1}$ is the most fundamental topic in statistics. Under this setting, the only covariate $V_{1}$ is the categorical population-ID taking values in {1,2}. The testing hypothesis problem and its subsequent ones can be turned into an equivalent problem: Is $V_{1}$ a major factor underlying the Re-Co dynamics of *Y*? If $V_{1}$ is not a major factor, then $H_{0}$ is accepted. If $H_{0}$ is indeed rejected by confirming $V_{1}$ being a major factor, then we would further want to discover where they are different.

For the illustrative simplicity, let $Y_{1j}∼N\left(0,1\right)$ and $Y_{1j}∼N\left(1,1\right)$ with j=1,…,N/2, that is, $N_{1}=N_{2}$. From a theoretical information measurement perspective, the theoretical value of entropy of *Y* is calculated being equal to H[Y]=1.5321, and its conditional entropy

$$H\left[Y|V_{1}\right]=\left(H\left[Y|V_{1}=0\right]+H\left[Y|V_{1}=1\right]\right)/2=\left(1.4189\times 2\right)/2=1.4189,$$

so the mutual information shared by *Y* and $V_{1}$ is denoted and calculated as $I\left[Y;V_{1}\right]=H\left[Y\right]-H\left[Y|V_{1}\right]=0.1132$. By $V_{1}$ being a major factor of *Y*, we mean that the $V_{1}$ is not replaceable by other covariate variables that is stochastically independent of *Y*, such as fair-coin-tossing random variable ε. That is, we theoretically establish this fact by knowing $0=I\left[Y;\varepsilon \right]<<I\left[Y;V_{1}\right]$.

In the real world, the two population-specific distributions $F_{1}\left(.\right)$ and $F_{2}\left(.\right)$ are often unknown. To accommodate this realistic setting, we build a histogram, say $\hat{F}\left(.\right)$, based on pooled observed dataset $\left\{Y_{ij}|i=1,2;j=1,\ldots ,N_{i}\right\}$. With a chosen version of $\hat{F}\left(.\right)$ with $K^{\prime }$ bins, we can build a $2\times K^{\prime }$ contingency table, denoted by $C[V_{1}-vs.-Y]$. Its two rows correspond to two population-IDs and all $K^{\prime }$ bins with column-sums $n_{k},k=1,\ldots K^{\prime }$ being arranged along the column-axis. That is, $C[V_{1}-vs.-Y]$ keeps the records of popultion-IDs for all members within each bin of $\hat{F}\left(.\right)$, and enable us to estimate the mutual information:

$$I\left[Y;V_{1}\right]=H\left[Y\right]-H\left[Y|V_{1}\right]=H\left[V_{1}\right]-H\left[V_{1}|Y\right].$$

All estimates of $I[Y;V_{1}]$ would be compared with estimates of I[Y;ε] from 2×K contingency tables generated as follows: its *k*th column with $k=1,\ldots ,K^{\prime }$ simulated from a binomial random variable $BN(n_{k},P_{0})$ with $P_{0}={\left(N_{1}/N,N_{2}/N\right)}^{\prime }$. This comparison of $I[Y;V_{1}]$ with I[Y;ε] is a way of testing whether a major factor candidate satisfies the criterion [C1: confirmable] in [15]. Precisely this testing is performed by comparing the observed estimate of $I[Y;V_{1}]$ with respect to the simulated distribution of I[Y;ε].

To make our focal issue concrete and meaningful, we undertake the following simulation study, in which the reliability issue of $H[Y|V_{1}]$ estimation is addressed, and at the same time [C1: confirmable] is tested. Recall that $Y_{1j}∼N\left(0,1\right)$ and $Y_{1j}∼N\left(1,1\right)$ with j=1,…,N/2. We consider two cases of N=2000 and N= 20,000. For practical considerations with respect to the infinity range of Normality, we choose $K^{\prime }=K+2$ bins for building a histogram via a 1+K+1 fashion. The observed 90% quantile range $\left[F_{N}^{-1}\left(0.05\right),F_{N}^{-1}\left(0.95\right)\right]$*K* is divided into *K* equal size of bins, while the first bin is $\left(-\infty ,F_{N}^{-1}\left(0.05\right)\right]$ and last bin is $\left[F_{N}^{-1}\left(0.95\right),\infty \right)$. We use 5 choices of K∈{10,20,30,100,1000}. For each *K* value, the estimated Shannon entropy $H^{\left(K\right)}\left[Y\right]$ and conditional entropies $H^{\left(K\right)}\left[Y|V_{1}\right]$. Also, a 95% confidence range (CR) of I[Y;ε] is also simulated and reported based on an ensemble of $I^{\left(K\right)}\left[Y;\varepsilon \right]=H^{\left(K\right)}\left[Y\right]-H^{\left(K\right)}\left[Y|\varepsilon \right]$, where ε is Bernoulli (fair-coin tossing) random variable.

As reported in the table Table 1, it is evident that the mutual information $I^{\left(K\right)}\left[Y;V_{1}\right]=H^{\left(K\right)}\left[Y\right]-H^{\left(K\right)}\left[Y|V_{1}\right]$ is very close to the theoretical values as if they are nearly scale-free when K=10,20,30 with N=2000 and K=10,20,30,100 with N= 20,000. The rule of thump in this 1D setting seems to be: the mutual information estimations are rather robust when the averaged cell count is over 30. When the average cell count is around 10, we begin to see the effects of finite sample phenomenon. Nonetheless, we still have estimates of $I^{\left(K\right)}\left[Y;V_{1}\right]$ being far above the upper limits of 95% confidence range of I[Y;ε] when K=100 with N=2000 and even K=1000 with N= 20,000. This simulation indeed points to an observation that the conclusion based on $I^{\left(K\right)}\left[Y;V_{1}\right]$ tends to rather reliable in view of [C1: confirmable] criterion.

In summary, Table 1 indicates that the estimate of the mutual information of $I[Y|V_{1}]$ is far above the 95% confidence range under the null hypothesis within each of all 5 choices of *K* under the two cases of *N*. 9 out of 10 cases have almost 0 *p*-values, except the 1+1000+1 case with N=2000. These facts indicate one common observation: when all bins contain at least 20 data point, the estimate of $I[Y|V_{1}]$ is reasonably stably and practically valid. That is, we only need a stable and valid estimate of $I[Y|V_{1}]$ for the purpose of confirming a major factor candidacy.

In fact, it is surprising to see that, even when K=1000 in the case of N=2000, $I^{\left(K\right)}\left[Y;V_{1}\right]$ still retains [C1: confirmable] criterion by going beyond the upper limit of the 95% confidence range of I[Y;ε]. This fact implies the correct decision is still being retained because $V_{1}$ is confirmed as a major factor. These observations become crucial when estimations of $I[Y|V_{1}]$ are facing the effects of the curse of dimensionality, also called finite sample phenomenon.

As $V_{1}$ being determined as a major factor underlying the dynamics of *Y* and the hypothesis $H_{0}$ is rejected, we then can check which of K+2 bins’ observed entropies fall inside or outside of bin-specific entropy-confidence-ranges built by simulated counts via $BN(n_{k},P_{0})$ across k=1,…,K+2. By doing so, we discover where $F_{1}\left(.\right)$ and $F_{2}\left(.\right)$ are different locally.

Next, one very interesting observation is found and reported in Table 1: values of $H^{\left(K\right)}\left[Y\right]$ vary with respect to *K*, but $I^{\left(K\right)}\left[Y;V_{1}\right]$ is nearly scale-free (w.r.t *K*). We explain how this observation occurs. Let $f\left(y\right)=F^{\prime }\left(y\right)$ be the hypothetical density function of random variable *Y* with observed values $\left\{Y_{lj}|l=1,2;j=1,\ldots ,N/2\right\}$. Based on fundamental theorem of calculus, for each *K*, we have the theoretical Shannon entropy $\tilde{H}\left(Y\right)$ is approximated as:

$$\begin{array}{ccc} H[Y] & = & \left(-1\right)\int_{-\infty }^{\infty }f\left(y\right)\log f(y)dy,\\ & ≅ & \left(-1\right)\sum_{k=0}^{K+1}f\left(y_{k}^{*}\right)\Delta (K)\log f(y_{k}^{*}),\\ & = & \left(-1\right)\sum_{k=0}^{K+1}p_{k}\log\frac{p_{k}}{\Delta (K)},\\ & = & H^{\left(K\right)}\left[Y\right]+\log\Delta (K), \end{array}$$

where $y_{k}^{*}$s denote inter-middle values in Mean Value Theorem of Calculus and $\Delta (K)=\frac{F_{N}^{-1}\left(0.95\right)-F_{N}^{-1}\left(0.05\right)}{K}$.

And we have

Δ(10)=JΔ(J×10).

with J=2,3,10 and 100. Therefore, we have the approximating relations as:

$$H^{\left(10\right)}\left[Y\right]\approx H^{\left(J\times 10\right)}\left[Y\right]-\log J.$$

After some subtractions, the differences are close to log2, log3, log10 and log100, which matches with numbers shown in the 3rd column of Table 1.

By the same reason, these relations hold for estimated conditional entropies as well. That is, we also have: for all *K*s,

$$H\left[Y|X\right]≅H^{\left(K\right)}\left[Y|X\right]+\log\Delta (K),$$

when all involving bins have 30 or so data points, as seen in 4th column of Table 1. This is the reason why that we see estimated values of $I^{\left(K\right)}\left[Y;V_{1}\right]$ being nearly constant (w.r.t *K*) when K=10,20,30 with N=1000 and K=10,20,30,100 with *N* = 10,000. This is a critical fact that we can employ mutual information estimates with reliability. Thus, we use the notation $I[Y;V_{1}]$ from here on, instead of $I^{\left(K\right)}\left[Y;V_{1}\right]$.

Here we further remark that the two-sample hypothesis testing problem (L=2) setting can be extended into the so-called multiple-sample problem (L>2). Correspondingly, categorical variable $V_{1}$ of population-IDs is equipped with *L* categories. This hypothesis testing:

$$H_{0}:F_{l}\left(y\right)=F\left(y\right),\;\forall y\in R^{1},l=1,\ldots ,L.$$

retains the same equivalent formulation as: Is $V_{1}$ a major factor underlying the dynamics of *Y*? This multiple-sample problem is also known as one-way ANOVA, which is one fundamental topic problem in Analysis of Variance.

Another fundamental topic problem in Analysis of Variance is represented by two-way ANOVA, which involved two categorical covariate features: $V_{1}$ and $V_{2}$. Let these two covariate features have $L_{1}$ and $L_{2}$ categories, respectively. Within a population with $V_{1}=l$ and $V_{2}=h$, measurements $Y_{lhj}$ are distributed with respect to $F_{lh}\left(.\right)$ with $l=1,\ldots ,L_{1}$ and $h=1,\ldots ,L_{2}$.

The classic two-way ANOVA setting is specified by assuming Normality distribution $Y_{lhj}\;N\left(\mu_{lh},\sigma^{2}\right)$ and $\mu_{lh}$ satisfying the following linear structure:

$$\mu_{lh}=\mu +\alpha_{l}+\beta_{h}+\gamma_{lh},$$

with μ as the overall effect, $\alpha_{l}$s the effects of $V_{1}$, $beta_{h}$s as effects of $V_{2}$, and $\gamma_{lh}$s as interacting effects of $V_{1}$ and $V_{2}$. These effects parameters are to satisfy the following linear constraints:

$$\sum_{l=1}\alpha_{l}=\sum_{h=1}\beta_{h}=\sum_{l=1}\gamma_{lh}=\sum_{h=1}\gamma_{lh}=0.$$

It is evident that this classic two-way ANOVA formulation is rather limited in the sense of excluding the possibility that $Y_{lhj}$ does not have an informative mean, such as non-normal distributions with heavy tails or more than one mode, or even lacking of the concept of mean, such as a categorical variable.

A much widely extended two-way version is given as follows:

$$F_{lh}\left(.\right)≅G\left[M_{1}\left(V_{1}\right),M_{2}\left(V_{2}\right),M_{12}\left(V_{1},V_{2}\right)\right],$$

where G[.] is unknown global function consisting of the following unknown component-wise mechanisms: the unknown component mechanism $M_{1}\left(V_{1}\right)$ having $V_{1}$ as its order-1 major factor; another unknown component mechanism $M_{2}\left(V_{2}\right)$ having $V_{2}$ as its order-1 major factor; and the unknown interacting component mechanism $M_{12}\left(V_{1},V_{2}\right)$ with $\left(V_{1},V_{2}\right)$ as its order-2 major factor. Our goal of data analysis under this extended version is again reframed as computationally determining whether these order-1 and order-2 major factors are present or not underlying the Re-Co dynamics of *Y* against the covariate features $V_{1}$ and $V_{2}$. If both covariate features $V_{1}$ and $V_{2}$ are independent or only slightly dependent with each other, the right major factor selection protocol can be found in [15]. However, if they are heavily associated, a modified major factor selection protocol can be found in [17].

We conclude this Example-1 with a summarizing statement: a large class of statistical topics can be rephrased and reframed into a major factor selection problem, and then this problem is resolved commonly by evaluating mutual information estimations that are not required to be precisely close to its unknown theoretical value.

### 2.2. [Example-2]: From Dealing to Lessening the Effects of Curse of Dimensionality

It is noted here that, mutual information $I[Y;V_{1}]$ has another representation

$$I\left[Y;V_{1}\right]=H\left[Y\right]+H\left[V_{1}\right]-H\left[Y,V_{1}\right]=\int_{R^{2}}dP\left(Y,V_{1}\right)\log\left\{\frac{dP(Y,V_{1})}{d(P\left(Y\right)\times P\left(V_{1}\right))}\right\}.$$

This presentation is valid even for a categorical variable $V_{1}$. Based on this representation, we can clearly see the scale-free property of mutual information with respect to various choices of histograms. Nonetheless, we refrain from using this definition for estimating $I[Y;V_{1}]$. Since this definition-based estimation involves the estimation of joint distribution of $\left(Y,V_{1}\right)$, which is a harder problem due to its dimensionality. This so-called curse of dimensionality would become self-evident later on in our developments when the response variable Y and its covariate features $\left(V_{1},\ldots ,V_{k}\right)$ are both multiple dimensional. The task of estimating multiple dimensional density becomes neither practical, nor reliable, given an ensemble of finite sample data points.

In this subsection, we demonstrate how to effectively deal with the effects of the curse of dimensionality. We consider again a two-sample problem, but having multiple dimensional data points, not single dimensional ones as in Example-1. Again we denote two populations with IDs: $V_{1}=0$ and 1. Data points from these two populations are denoted as $Y^{0}=\left(Y_{1}^{0},\ldots ,Y_{m}^{0}\right)$ and $Y^{1}=\left(Y_{1}^{1},\ldots ,Y_{m}^{1}\right)$ with m>1, respectively. Let $Y=(Y_{1},\ldots ,Y_{m})$ denote the multiple dimensional response variable. To resolve the same task of testing whether these two populations are equal with *m* components possibly highly associative features, what would be the best way of building up the contingency table for the purposes of estimating the $I[Y;V_{1}]$ for testing the hypotheses?

We expect the equal-bin-size and equal-bin-area approaches for component-wise histograms are neither ideal nor practical due to the curse of dimensionality. On the other hand, we know that the clusters of *m*-dim data points can naturally retain the dependency structures. Hence, it is intuitive to employ results of clustering algorithms to differentiate patterns of structural dependency within $Y^{0}$ and $Y^{1}$. This intuition leads to the important merit of cluster-based contingency table as a way of lessening effects from the curse of dimensionality. We illustrate these ideas through two samples of simulated multivariate Normal-distributed data described as follows.

Let m=4 and two mean-zeros Normal distributions: $Y^{0}∼N\left(\tilde{0},\Sigma^{0}\right)$$Y^{1}∼N\left(\tilde{0},\Sigma^{1}\right)$.

$$\Sigma^{0}=\left[\begin{array}{cccc} 1 & \rho^{0} & \rho^{0} & \rho^{0}\\ \rho^{0} & 1 & \rho^{0} & \rho^{0}\\ \rho^{0} & \rho^{0} & 1 & \rho^{0}\\ \rho^{0} & \rho^{0} & \rho^{0} & 1 \end{array}\right],\Sigma^{1}=\left[\begin{array}{cccc} 1 & \rho^{1} & \rho^{1} & \rho^{1}\\ \rho^{1} & 1 & \rho^{1} & \rho^{1}\\ \rho^{1} & \rho^{1} & 1 & \rho^{1}\\ \rho^{1} & \rho^{1} & \rho^{1} & 1 \end{array}\right]$$

The Shannon entropies of these two 4D Normal distributions via the following formula with d=4:

1/2log(det(Σ))+d/2(1+log(2π))

are calculated as 5.0942 and 4.4355, respectively. So the $H\left[Y|V_{1}\right]=\left(5.0942+4.4355\right)/2=4.7648$. As for H[Y] of the mixture of two 4D Normal distributions, its calculation is not straightforward and even troublesome. Through an extra experiment using 100 millions of data points, we end with a negative estimate of the mutual information. This failed attempt in fact further provides a vivid clue of the effect of curse of dimensionality. In other words, we need to resolve such an effect by staying away from the rigid 4D hypercubes.

**Figure 1:**
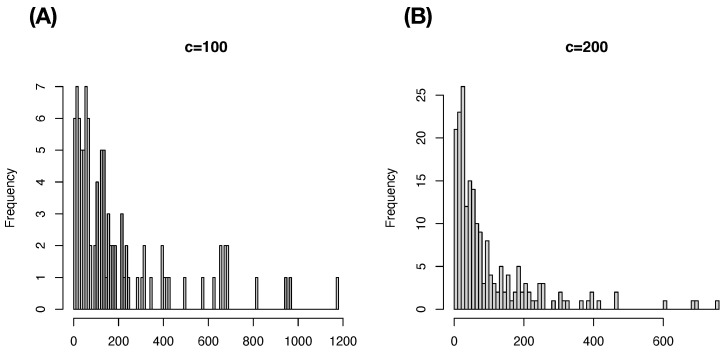
Comparing Hierarchical clustering and K-means via distributions of cluster sizes in Example 2: (**A**) c=100; (**B**) c= 200.

**Figure 2:**
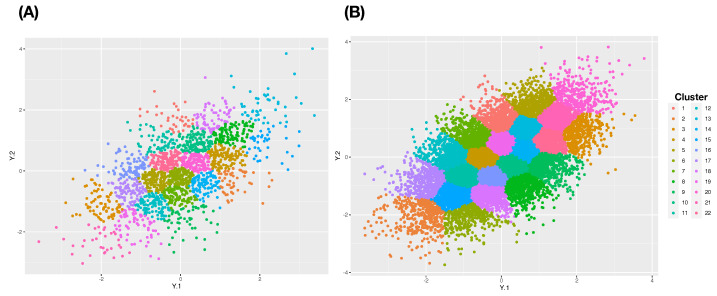
K-mean clusters in 2D setting: (**A**) N=2000; (**B**) N= 20,000.

In contrast, we demonstrate that the cluster-based approaches are potentially reasonable choices to mend this effect of the curse of dimensionality. Consider two commonly used clustering algorithms: Hierarchical clustering (HC) and K-means algorithms. It is also known that the HC algorithm is computationally more costly than the K-means algorithm. Since the HC-algorithm heavily relies on a distance matrix, HC-algorithm has difficulties in handling a data set with a very large sample size. Recently, very effective computing packages have been developed for K-means algorithm, that is, K-means algorithm can be effectively applied. On top of computing efficiency differences, there exists a critical difference between the two algorithms. The K-means provides much more even cluster-sizes than HC-algorithm does as illustrated in Figure 1, see also Figure 2. For these reasons, we employ K-means clustering, not Hierarchical clustering (HC), algorithm in the following series cases with m=2,3,4.

In this experiment, we take $\rho^{0}=0.5$ and $\rho^{1}=0.7$ under two settings with N=2000 and N= 20,000. It is noted that the differences in $\rho^{0}$ values imply the differences in distribution shapes. The series of clustering compositions are constructed as follows. We apply the K-means algorithm to derive a series of clustering compositions with 12, 22, 32 and 102 clusters. Correspondingly, we built a series of contingency tables of the formats: (1) 2×12; (2) 2×22; (3) 2×32 and (4) 2×102. With respect to the series of clustering compositions, we compute H[Y] and $H[Y|V_{1}]$ and $I[Y;V_{1}]$. Here, $V_{1}$ is again the categorical variable of population-IDs.

The messages derived from Example-1 are also observed in Example-2 across 2D to 4D settings in Table 2, Table 3 and Table 4. These results clearly indicate that distribution shape differences can be effectively and reliably picked up by entropy-based evaluations of mutual information between the *Y* and categorical label variable $V_{1}$. These results imply that we widely extend one-way ANOVA and two-way ANOVA settings to accommodate high dimensional data points as we have argued in Example-1.

**Figure 3:**
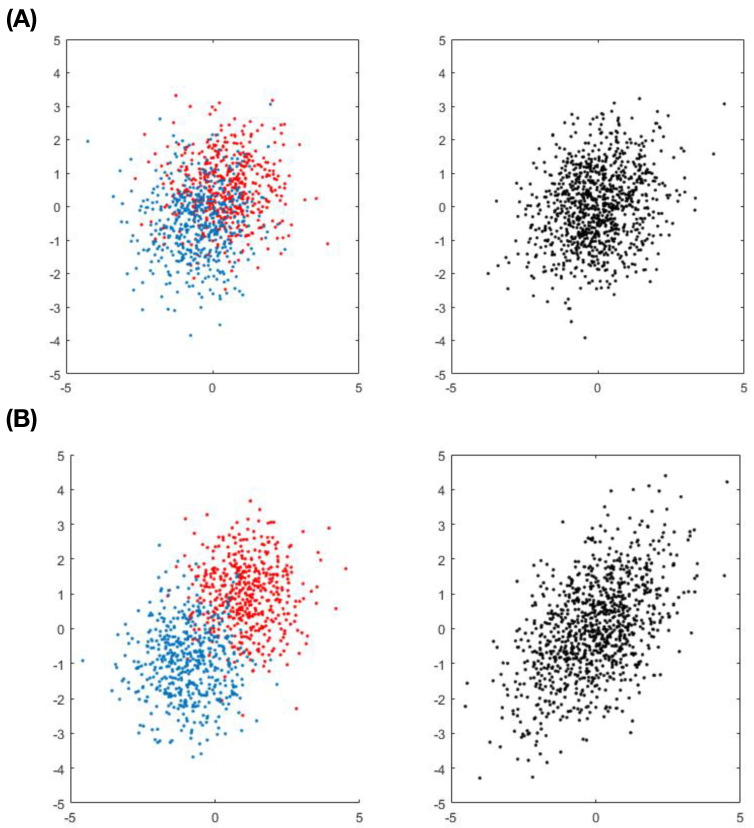
Two sets of pairwise scatter-plots of one simulated 2D normal mixture against 2D normal with equal mean vector and covariance matrix. (**A**) The first set is for two close normal mixture with mean vectors: (0.5,0.5) and (−0.5,0.5) and (**B**) the second is for relative apart normal mixture.

In order to better understand the limit of such an entropy-based approach, we twist the 2D setting in Example-2 a little bit. This more complicated version of Example-2, denoted as Example-$2^{*}$, consists of one 2D normal mixture and one 2D normal. These two 2D distributions are further made to have equal mean vector and covariance matrix. Furthermore, two kinds of mixture-settings are designed and used. The first setting of Example-$2^{*}$ is designed for a mixture of two relatively close 2D normals with mean vectors: (0.5,0.5) and (−0.5,0.5). The second setting is designed for a relatively apart normal mixture with mean vectors: (−1,−1) and (1,1). These two settings of pairwise scatter-plots are given in Figure 3. It is obvious that we can visually separate the two 2D distributions in the second mixture setting, but can not do equally well in the first mixture setting.

The mutual information estimates and confidence ranges under the null hypothesis are calculated and reported in Table 5. In the first mixture setting, it is apparent that $V_{1}$ fails to be a major factor by failing to satisfy the criterion [C1: confirmable] across all *K* choices. This result is coherent with our visualization through the upper panel Figure 3. As for the 2nd mixture setting, $V_{1}$ is claimed as a major factor by satisfying the [C1: confirmable] criterion across all *K* choices. This result is also coherent with our visualization through the lower panel Figure 3. Further, we observe that the relative position of I[Y;X] estimates against upper and lower limits of null confidence ranges are rather stable when the sizes of clusters are not too small. This observation indeed provides us with the practical guideline for varying choices of *K* according to different sample sizes when we employ mutual information to perform inferences under Re-Co dynamics.

We conclude this Example-2 (Example-$2^{*}$) with a summarizing statement: Though, any theoretical evaluations of mutual information under the presence of high dimensionality are practically impossible, clustering algorithms provide practical guidelines for building contingency tables and evaluating mutual information for inferential purposes by lessening the effects of curse of dimensionality.

### 2.3. [Example-3]: From Linear to Highly Nonlinear Associations

We then turn to consider the simplest one-sample problem involving dependent 2D data points. The framework of Re-Co dynamics is self-evident. In this example, we examine the validity and performances of inferences based on estimated mutual information between two 1D continuous random variables *Y* and *X* via contingency tables of various dimensions. For simplicity in the first scenario of Example-3, we consider a bivariate normal $\left(Y,X\right)∼N\left(\tilde{0},\Sigma \right)$ with covariance matrix:

$$\Sigma =\left[\begin{array}{cc} 1 & \rho \\ \rho & 1 \end{array}\right],$$

Here the correlation coefficient ρ is taken to be 0.0 and 0.5, respectively, in this experiment with N=1000 or 10,000. The contingency tables are derived from the K-means algorithm being applied on *X* and *Y*, respectively, with a series of pre-determined numbers of clusters: {12,22,32,102}.

For the setting of ρ=0, we report the calculated I[Y;X] and confidence range of I[Y;ε] in Table 6 across the 16 dimensions of contingency tables. The smallest size of the contingency table has 144(=12×12) cells. Its average cell-count is less than 14 for N=2000. The largest size of the contingency table is 102×102, which is more than $10^{4}$. Its averaged cell-count is less than 2 for N= 20,000.

From the upper half of Table 6 for the N=2000, all estimates of I[Y;X] are beyond the upper limit of 95% confidence range of I[Y;ε]. That is, the hypothesis of *Y* and *X* being independent is falsely rejected. In contrast, from the lower half of Table 6 for the N= 20,000, all estimates of I[Y;X] are either below the lower limit of 95% confidence interval of I[Y;ε] or within confidence range, except the results based on the largest 102×102 contingency table. That is, the same independence hypothesis would not be falsely rejected except in the case of the largest contingency table. Such a contrasting comparison between the upper and lower halves of Table 6 clearly indicates that validity of mutual information evaluations heavily rely on degrees of volatility of cell counts, especially on testing independence. We further explicitly express such volatility below.

A simple reasoning for the above results goes as follows. For this independent setting of *Y* and *X*, for expositional simplicity, let all cells in contingency tables have equal probability. In the smallest contingency table, the cell probability is 1/144. The cell-count is a random variable with mean and variance being very close to N/144 as well. Thus, the cell-count is falling between $N/144\pm 2\sqrt{N/144}$ with at least 95%. With N=2000, the 95% range is close to [6,22], while with N= 20,000 the 95% range is close to [110,150]. Based on these two 95% intervals, we can see that the Shannon entropy along each row of the 12×12 contingency table can be volatile with N=2000, while it is not the case with N= 20,000. In fact, when N=2000, a 6×6 contingency table indeed provides much more stable evaluations of mutual information.

In the setting of ρ=0.5, we report the calculated I[Y;X] and confidence range of I[Y;ε] in Table 7 across the 16 dimensions of contingency tables with N= 20,000. We observe that the calculated I[Y;X] is far above the upper limit of the confidence interval of I[Y;ε] even in the largest contingency table with dimension 102×102. The reason is that the number of effectively occupied cells are much smaller due to the dependency, that is, many cells supposed to be empty are indeed empty. With many empty cells coupling with many occupied cells with relatively large cell counts, the Shannon entropy is evaluated with great stability. These results from independent and dependent experimental cases are learned to constitute practical guidelines for evaluating mutual information.

**Figure 4:**
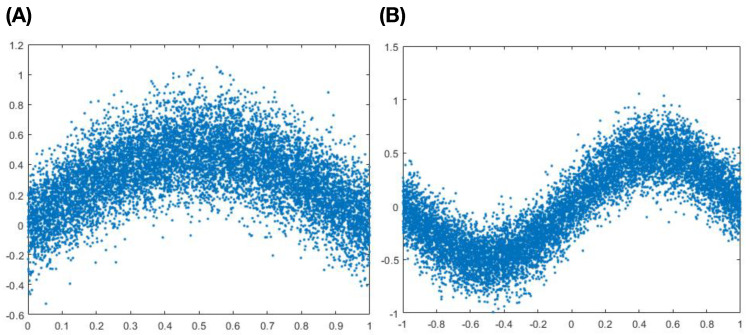
Scatter-plots of two simulated data sets in sine functional shapes: (**A**) half-sine function; (**B**) full-sine function.

The second scenario of Example-3 is about whether the calculated mutual information I[Y;X] can reveal the existence of non-linear association between *Y* and *X*. We generate two simulated data sets based on two non-linear associations: (1) half-sine function; (2) full-sine function, as shown in the two panels of Figure 4. Within both cases of non-linear associations, it is noted that the correlations of *Y* and *X* are basically equal to zero.

In the setting of a half-sine functional relation, we report the calculated I[Y;X] and confidence range of I[Y;ε] in Table 8 across the 16 dimensions of contingency tables with N= 20,000. Across all 16 dimensions of contingency tables, the calculated I[Y;X] are far beyond the upper limits of confidence intervals of I[Y;ε]. As far as p-value being concerned, they are all basically zeros. The same results are observed in the setting of full-sine functional relations as reported in Table 9. These two settings in this non-linear association scenario together demonstrate that the calculated I[Y;X] can reveal the existence of significant association between *Y* and *X*. This demonstration is important in the sense of without knowing the functional forms of their association.

We summarize the practical guidelines that we learned from Example-1 through Example-3 in this section. The most apparent fact is that the calculated values of mutual information I[Y;X] vary with respect to dimensions of contingency tables. However, the good news is that the amounts of variations are relatively small and even very minute when cell-counts in the contingency table are not too low. Nonetheless, the calculated mutual information I[Y;X] is very capable of revealing the presence and absence of associations underlying Re-Co dynamics of response variable *Y* and covariate variable *X* from the three examples and scenarios considered in this section. And it is a reliable way of seeking consistent inferential decisions by varying contingency tables’ dimensions. This capability can be made very efficient if we choose the dimension of the contingency table to suitably reflect the total sample size of the data set with varying degrees. That is, we make sure such efficiency is achieved by varying the dimensions of contingency tables from small to reasonably large. The final guideline is that comparability between two mutual information evaluations is resting on their more or less identical computational platforms, that is, their contingency tables are more or less the same in dimensions. On the other hand, the averaged numbers of cell counts are relatively large, and mutual information evaluations are rather robust to some degree of differences in contingency tables’ dimensions. These practical guidelines will ascertain mutual information evaluations always coupled with reliability. Finally, the data-types of *Y* and *X* are entirely free because we rely on their categorical nature only.

## 3. Examples with Complex Re-Co Dynamics

Next, we consider two examples with Re-Co dynamics wich are more complex than the three examples discussed in the previous section. Through these two examples that havie independent covariate features, we further illustrate the necessity of following the practical guidelines motivated and learned in the previous section.

### 3.1. [Example-4]: From Complex Interaction to Further Beyond

After going through three relatively simple examples in the previous section, we now turn to examples with more complex Re-Co dynamics. Consider a functional relation between *Y* and $\left\{X_{1},\ldots X_{4}\right\}$ specified as follows:

$$Y=X_{1}+\sin\left(2\pi \left(X_{2}+X_{3}\right)\right)+N\left(0,1\right)/10$$

with $\left\{X_{1},\ldots X_{4}\right\}$ being i.i.d. U[0,1] and N= 10,000. That is, $X_{4}$ plays the role of observable noise random variable, while unobservable noise is N(0,1)/10. Our goal is to discover the order-1 major factors $X_{1}$ and order-2 major factor $\left(X_{2},X_{3}\right)$. It is worth noting that this order-2 major factor can not be discovered via linear regression analysis, even when the product type of interacting effect is included in the model.

The response variable *Y* is categorized with 12 bins, so does each of the 4 covariate features. We calculate mutual information of *Y* and all possible feature subsets’ $A\subseteq \{X_{1},\ldots X_{4}\}$, say I[Y;A]. If |A|=k, we build a ${\left(12\right)}^{k}\times 12$ contingency table for calculating for evaluating I[Y;A]. Here *A* also stands for a fused categorical variable in the sense that categories of *A* are all occupied *k*D hypercubes of its k(=|A|) feature-members.

We compute and report conditional entropies (CEs) for all possible *A*s and arrange them with respect to sizes |A| of *A* in Table 10. Also we report a term called successive (S) CE-drops defined via the following CEs difference:

$$SCE_{drop}\left[Y|A\right]=\left(H\left[Y\right]-H\left[Y|A\right]\right)-\max_{A^{\prime }\subset A}\left\{H\left[Y\right]-H\left[Y|A^{\prime }\right]\right\}=\min_{A^{\prime }\subset A}\left\{H\left[Y|A^{\prime }\right]\right\}-H\left[Y|A\right].$$

This SCE term is designed to evaluate the extra effect of CE-drop by including an extra feature-member. The above formula is precise in theory. But in reflecting the aforementioned last practical guideline in the last section, it is essential to note that SCE[Y|A] involves at least two different settings of |A|=k and $|A^{\prime }|=k^{\prime }\left(<k\right)$, which correspondingly involve two different dimensions of contingency tables: one is of ${\left(12\right)}^{k}\times 12$ and the other is ${\left(12\right)}^{k^{\prime }}\times 12$. Therefore, based on what we have learned from the previous section, these settings render different scales of conditional entropy and mutual information computations. That is, these different scales will certainly make mutual information evaluations not completely comparable, especially when cell-counts in the contingency tables are overall too small. For instance,

$$SCE_{drop}\left[Y|X_{1},X_{2}\right]=0.0644=H\left[Y|X_{1}\right]-H\left[Y|X_{1},X_{2}\right]=2.2315-2.1671.$$

The SCE-drop of $\left(X_{1},X_{2}\right)$ is more than 10 times of CE-drop of $X_{2}$. It would be a mistake to claim that $X_{1}$ and $X_{2}$ are conditional dependent given *Y*. Since the scale in evaluating $H[Y|X_{1}]$ is different from the scale in evaluating $H[Y|X_{1}X{,}_{2}]$. Nevertheless, since $X_{4}$ plays a role of random noise in this example, the information contents of $X_{1}$ and $\left(X_{1},X_{4}\right)$ are supposed to be very close from the perspective of their contingency table. Theoretically, we have $H\left[Y|X_{1}\right]=H\left[Y|X_{1},X_{4}\right]$. That is, $H[Y|X_{1},X_{4}]$ should represent the information content of $X_{1}$ upon the setting of ${\left(12\right)}^{2}\times 12$ contingency table. Along this line of argument, we should refine the SCE-drop as follows:

$$SCE_{drop}^{*}\left[Y|X_{1},X_{2}\right]=H\left[Y|X_{1},X_{4}\right]-H\left[Y|X_{1},X_{2}\right]=2.1685-2.1671=0.0014.$$

Using the same argument, this SCE-drop should be compared with $H\left[Y|X_{4}\right]-H\left[Y|X_{2},X_{4}\right]=2.4557-2.3780=0.0777$, which is 5 times larger than 0.0014. Hence, it is obvious that $X_{1}$ and $X_{2}$ do not have joint interacting effects. In fact, it would be more precise evaluation of the effect of $X_{2}$ under the 2-feature setting if we use $H\left[Y|X_{4},X_{5}\right]-H\left[Y|X_{2},X_{4}\right]$ with $X_{5}$ being another irrelevant independent U[0,1] random variable. However, according to the guidelines learned from example-1 and -2, $H[Y|X_{4},X_{5}]$ and $H[Y|X_{4}]$ should be relatively close because of the sample size of 10,000.

This line argument ultimately converges to the following practical guideline on evaluating Information Theoretical measurements via contingency table platform: “these CEs and mutual information measurements are comparable only when they are evaluated under the same dimensions of contingency tables”. This guideline indeed is coherent with a statistical concept of conditioning with respect to the observed row-sum vector.

Before summarizing our findings from Table 10, where we reported calculated CEs and $SCE_{drop}$, we need to prepare baseline-evaluations to make sure that all CEs comparisons are sensible. Here, we recall that C[A−vs.−Y] denotes the contingency table with categories of *Y* on column-axis and categories of covariate feature subset *A* on row-axis.

- **1-feature setting:** With $C[X_{1}-vs.-Y]$ having its proportion vector of row-sums denoted as $P_{X_{1}}$, we build an ensemble of $C[X_{1}^{\varepsilon }-vs.-Y]$ by distributing *i*-th column-sum N[Y=i] with respect to $Multinomial(N\left[Y=i\right],P_{X_{1}})$. The average of the CEs of $H[Y|X_{1}^{\varepsilon }]$, denoted as $E[H\left[Y|X_{1}^{\varepsilon }\right]]$ is designed to be comparable with $H[Y|X_{1}]$. Their difference $E\left[H\left[Y|X_{1}^{\varepsilon }\right]\right]-H\left[Y|X_{1}\right]$ is a proper and valid measurement of the CE-drop of $X_{1}$. Likewise for the remaining covariate features.
- **2-feature setting:** With $C[Y;\left(X_{1},X_{2}\right)-vs.-Y]$, we need to compute $E[H\left[Y|{\left(X_{1},X_{2}\right)}^{\varepsilon }\right]]$ for the joint CE-drop of $\left(X_{1},X_{2}\right)$ calculated as $E\left[H\left[Y|{\left(X_{1},X_{2}\right)}^{\varepsilon }\right]\right]-H\left[Y|\left(X_{1},X_{2}\right)\right]$. We also need $E[H\left[Y|\left(X_{1},X_{2}^{\varepsilon }\right)\right]]$ for calculating $SCE_{drop}^{*}\left[Y|X_{1},X_{2}\right]$ in order to be able to compare to $E\left[H\left[Y|{\left(X_{1},X_{2}\right)}^{\varepsilon }\right]\right]-E\left[H\left[Y|\left(X_{1}^{\varepsilon },X_{2}\right)\right]\right]$ to figure out the amount $I\left[\left(X_{1},X_{2}\right)|Y\right]-I\left[\left(X_{1},X_{2}\right)\right]$.
  As for $\left(X_{2},X_{3}\right)$, in comparison with SCEs of $\left(X_{2},X_{4}\right)$ and $\left(X_{3},X_{4}\right)$, its $SCE_{drop}$ is calculated as 0.7781, which is more than 10 times of $X_{3}$’s individual $SCE_{drop}$. This is a very strong indication of the interacting effect of $\left(X_{2},X_{3}\right)$ due to evident presence of their conditional dependency given *Y*. This fact establishes the feature-pair $\left(X_{2},X_{3}\right)$ as an order-2 major factor.
- **3-feature setting:** In Table 10, the $SCE_{drop}$ of feature-triplet $\left(X_{1},X_{2},X_{3}\right)$ from feature-pair $\left(X_{2},X_{3}\right)$ is 0.8431, which is about 3.5 times of CE-drop of $X_{1}$. This observation could seemingly point to the potential presence of conditional dependency of $\left(X_{1},X_{2},X_{3}\right)$. However, if we more precisely calculate the effect of $X_{1}$ when adding to $\left(X_{2},X_{3}\right)$ as:
  $$SCE_{drop}^{*}\left[Y|X_{1},X_{2},X_{3}\right]=H\left[Y|X_{2},X_{3},X_{4}\right]-H\left[Y|X_{1},X_{2},X_{3}\right]=1.2263-0.8362=0.3901,$$
  and compare it with $H\left[Y|X_{4},X_{5},X_{6}\right]-H\left[Y|X_{1},X_{4},X_{5}\right]$ with $X_{5}$ and $X_{6}$ being independent random variables, which is expected to be larger than 0.2322, but smaller than 0.3901. Therefore, we can only confirm that the ecological effect does exist between $X_{1}$ and $\left(X_{2},X_{3}\right)$, that is, they can be order-1 and order-2 major factors of *Y*. But, certainly they don’t form conditional dependency underlying *Y*, see details of major factor selection protocol in [15].

### 3.2. [Example-5]: From High-Order Interaction to Complexity

In order to see the effect of higher order major factor, we change the functional form of *Y* slightly as:

$$Y=X_{1}+\sin\left(2\pi \left(X_{2}+X_{3}+X_{4}\right)\right)+N\left(0,1\right)/10.$$

With sample size *N* = 10,000, our computational results are reported in Table 11. Likewise, we can confirm $X_{1}$ as an order-1 major factor and triplet $\left(X_{2},X_{3},X_{4}\right)$ as an order-3 major factor. In sharp contrast, the evidence of order-3 major factor seems to disappear when N=1000, as shown in Table 12. This is the exact demonstration of the effect of finite sample phenomenon, or curse of dimensionality. Do these two contrasting results: presence and absence of order-3 major factor in *N* = 10,000 and N=1000, respectively, mean that we should give up looking for high order major factors on small data sets?

The answer to the above question is negative. That is, somehow we can escape from the curse of dimensionality in our pursuit of high order major factor. Here we demonstrate a way of escaping. We perform K-means clustering on the 3D data points of $\left(X_{2},X_{3},X_{4}\right)$ with 12, 36, 72 and 144 clusters, with which we build a new covariate feature $X_{234}$. The CEs of $X_{234}$ with respect to the four corresponding contingency tables are reported in Table 13 with *Y* being categorized in 12 and 32 categories (clusters) via K-means. In the case of 12 clusters on *Y*, we see that the CE of $X_{234}$ is increasing from 20 to 60 standard deviations (sd) away from the mean CE of $X_{234}^{\varepsilon }$ as the numbers of clusters of $X_{234}$ increases from 12 to 144. We observe similar evidence in the case of 32 categories on *Y*.

We can then confirm $X_{234}$ as a new order-1 major factor, which is a condensed version of $\left(X_{2},X_{3},X_{4}\right)$. Therefore, we should also claim that $\left(X_{2},X_{3},X_{4}\right)$ is indeed an order-3 major factor. This is an important and significant demonstration that we can be sure about the presence of high order major factors even when the sample size is relatively low, that is, the curse of dimensionality is escapable.

Further, by contrasting Table 13 with Table 12, the biases of mutual information estimates indeed can be managed by reducing the large number of bins, cells or hypercubes on the covariate side. That is, a small number of clusters can be derived via a clustering approach of choice.

## 4. Examples with Complex Re-Co Dynamics with Dependent Covariate Features

In this section, we conduct one experimental Re-Co dynamics defined by linear structures with slightly dependent covariate features as specified below. That is, this experiment is in the classic linear regression domain. However, there are two twists included in this experiment. The first twist is that there exist two almost-collinearity 3D hyper-planes pertaining to two triplets of covariate features. The second twist is that, when a continuous measurement data type is altered into a categorical one, we understand that we discard very fine scale information of measurements often together with some degrees of ordinal relational information. Nevertheless, this act of investment by sacrificing some information in data is necessary for carrying out our CE computations in its quest for critical authentic information content contained in data. On the other hand, it is natural to ask the following question: When linear regression analysis is applied to such a categorized data set, do we naturally expect its conclusions from such an analysis to be close to the true linear structure?

In this section, we investigate the aforementioned two twists in order to understand the general effects of dependence on conditional entropy evaluations, and we also address the above question. The particular focuses are placed on issues linking to validity of Information Theoretical measurements and their reliability evaluations. We would like to demonstrate the comparisons between classical statistics and CEDA’s major factor selection upon the quests into Re-Co dynamics.

### 4.1. [Example-6]: From Dependency Induced Complications to Reality

Consider a Re-Co dynamics defined by linear structures with slightly dependent covariate-features:

$$\begin{array}{ccc} Y & = & X_{1}+X_{2}+X_{3}+N\left(0,1\right)/10,\\ X_{6} & = & (X_{1}+X_{2}+X_{3}+X_{4}+X_{5}+N\left(0,1\right)/10)/3,\\ & & \left(X_{1},\ldots ,X_{5},X_{7},\ldots ,X_{10}\right)∼N\left(\tilde{0},\Sigma \right),\\ \Sigma [i,i] & = & 1,\Sigma [i,j]=0.2,\;i\neq j,\;i,j\in \{1,\ldots ,9\}. \end{array}$$

where Σ is a 9×9 covariance matrix (not including $X_{6}$). Features $\left\{X_{7},X_{8},X_{9},X_{10}\right\}$ play the roles of unrelated, but dependent noise. The design of this Example-6 is to have a seemingly dominant order-1 major factor candidate: feature $X_{6}$. We want to explore whether we could discover the true structure underlying the RE-Co dynamics that is a collection of 3 order-1 major factors: $\left\{X_{1},X_{2},X_{3}\right\}$, or not. Also we would like to see what realistic computational issues are generated from the dependency among all covariate features.

One million 11dim data points are simulated and collected as the data set. We apply our CE computations by having all 1D covariate features and the response features are categorized to have 22 bins via the same scheme used in the previous section. CEs are calculated for all possible feature-sets via the contingency table platform. For expositional purposes, we only report 10 CE-values for 10 key characteristic feature-sets across 1-feature to 6-feature settings in Table 14. The summary of our findings based on major factor selections are reported below.

1. On 1-feature setting, $X_{6}$ has the lowest CE and members of $\left\{X_{1},X_{2},X_{3}\right\}$ are in the second tier by having the median tier of CEs, while the rest of covariate features are in the 3rd tier having the highest CEs. Therefore, each member of $\left\{X_{1},X_{2},X_{3},X_{6}\right\}$ is a potential order-1 major factor candidate. It is noted that, though H[Y]=3.0316 in the 0-feature setting, it is more proper to use $H^{\left(1\right)}\left[Y\right]=H\left[Y|X_{10}\right]=2.9883$ on 1-feature setting due to the contingency tables’ dimension-change from 1×22 to 22×22, as we have argued in the previous two sections.
2. On 2-feature setting, we take $H^{\left(2\right)}\left[Y\right]=H\left[Y|X_{4},X_{7}\right]=2.9523$ and calculate the CE-drop of $(X_{4},X_{6})=2.9523-2.1321=0.8202$ and CE-drop of $X_{6}$ as $H^{\left(2\right)}\left[Y\right]-H\left[Y|X_{6},X_{7}\right]=2.9523-2.3309=0.6214$. Since the CE-drop of $X_{4}$ is basically zero. So we know that $X_{6}$ and $X_{4}$ are potentially conditional dependent given *Y*, so are $X_{6}$ and $X_{5}$. Likewise, we calculated CE-drops of $\left(X_{6},X_{1}\right)$ and $X_{1}$ as 0.7084 and 0.2513. Thus, the CE-drop of $\left(X_{6},X_{1}\right)$ is smaller than the sum of CE-drops of $X_{6}$ and $X_{1}$. This is the first evidence that $X_{6}$ and any individual members of $\left\{X_{1},X_{2},X_{3}\right\}$ can not be order-1 major factors, simultaneously.
  In contrast, the CE-drop of $\left(X_{1},X_{2}\right)$ is calculated as 0.6338, which is only slightly larger than the sum of CE-drops of $X_{1}$ and $X_{2}$: 0.5026. This evidence of so-called ecological effect indicates that $X_{1}$ and $X_{2}$ are not significantly conditional dependent, but they can be order-1 major factors simultaneously. Likewise for $X_{1}$ and $X_{3}$ and $X_{2}$ and $X_{3}$.
3. On 3-feature setting, we take $H^{\left(3\right)}\left[Y\right]=H\left[Y|X_{7},X_{8},X_{9}\right]=2.8139$ and calculate the CE-drops of $\left(X_{1},X_{2},X_{3}\right)$ and $\left(X_{4},X_{5},X_{6}\right)$ as: 2.0596 and 1.7393, respectively. Though these two CE-drops are more than 3 times of the sums of individual CE-drops of these two triplets, which are 0.6870 and 0.5567, respectively, we do not claim that the two triplets $\left(X_{1},X_{2},X_{3}\right)$ and $\left(X_{4},X_{5},X_{6}\right)$ are potential candidates of order-3 major factors. Since there is no conditional dependency claims among members of these triplets in the 2-feature setting. However, we claim that $\left(X_{1},X_{2},X_{3}\right)$ is the chief collection of 3 order-1 major factors, while $\left(X_{4},X_{5},X_{6}\right)$ is an alternative collection of 3 order-1 major factors.
4. On 4-feature setting, we take $H^{\left(4\right)}\left[Y\right]=H\left[Y|X_{7},X_{8},X_{9},X_{10}\right]=1.6278$, which is significantly smaller than $H^{\left(3\right)}\left[Y\right]$. As expected, this is an evidence of effect of curse of dimensionality. Since the averaged cell count is less than 1 in this setting. Therefore, we can not make any structural claims here. (It is also reasonable to expect that, if the number of bins is reduced to 10, the 4-feature setting might yield stable evaluations of mutual information.)
5. On 5-feature and 6-feature settings, no creditable claims can be made due to curse of dimensionality.

Our conclusion in the 3-feature setting: the chief collection of order-1 major factors $\left\{\left(X_{1},X_{2},X_{3}\right)\right\}$ and one secondarily alternative collection $\left\{\left(X_{4},X_{5},X_{6}\right)\right\}$, is a unusual, but precise statement. This statement is in sharp contrast with classic regression analysis. For instance, for comparison purpose, we perform LASSO regressions, which is specified in the following Lagrangian form:

$$\min_{\beta \in R^{11}}{\{∥Y-X\beta ∥}_{2}^{2}+{\lambda ∥\beta ∥}_{1}\}.$$

**Figure 5:**
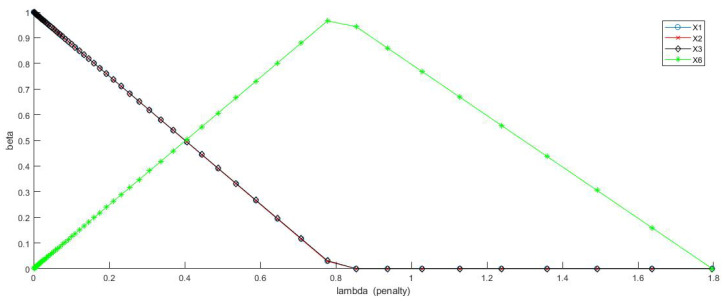
Results of parameters in Example-6 via LASSO with respect to a spectrum of λ penalty values. The three cures of $X_{1}$, $X_{2}$ and $X_{3}$ are completely overlapping with each other.

As shown in Figure 5, the joint presence of $\left\{X_{1},X_{2},X_{3},X_{6}\right\}$ are seen for all λ falling within (0,0.8). Specifically, the observed pattern is that parameters of members of $\left\{X_{1},X_{2},X_{3}\right\}$ are linearly decreasing from 1, while parameter of $X_{6}$ is increasing from 0 also linearly. Such linearity is primarily due to the penalty λ. All such trajectories of beta are not correct for the Re-Co dynamics except when λ=0, which only reports the result regarding $\left\{X_{1},X_{2},X_{3}\right\}$, but not $\left(X_{4},X_{5},X_{6}\right)$.

We conclude that, though the LASSO with manmade penalty constraints seemingly coupled with some desirable interpretations, its optimization protocol clearly can not handle a landscape having two equally probable “deep-wells”. In sharp contrast, our major factor selection protocol has no problems at all in identifying and confirming two collections of three order-1 major factors, and these two collections can not co-exist. This result is reiterated in the next subsection as well. This capability is the chief merit of employing Information Theoretical measures in major factor selection.

Further, we conduct the least squares estimation based on all categorized data, and report the results in Table 15. We can see that the results of estimations give rise to mixed-up and wrong linear structures. That is, the categorizing scheme, which heterogeneously alters locations and scales of original data, has indeed destroyed data’s intrinsic characteristics. From this perspective, we understand that the categorical nature of data is suitable for Information Theoretical Measures, but not for linear regression models and its variants.

### 4.2. Escaping from the Curse of Dimensionality

In Example-6, the 6-feature setting, the feature-set $\left\{\left(X_{1},X_{2},X_{3},X_{4},X_{5},X_{6}\right)\right\}$ achieves the largest CE among all possible feature-sets, which is at least 7 times of CE of

$\left\{\left(X_{1},X_{2},X_{3},X_{7},X_{8},X_{9}\right)\right\}$. Such comparisons are invalid due to finite sample phenomenon or curse of dimensionality. Since there are more than 1.408 billions (${\left(22\right)}^{7}$) 7D hypercubes for just one million data points. How can we escape from the potential effects of curse of dimensionality on estimations of CEs of $\left\{\left(X_{1},X_{2},X_{3},X_{4},X_{5},X_{6}\right)\right\}$ and $\left\{\left(X_{1},X_{2},X_{3},X_{7},X_{8},X_{9}\right)\right\}$?

Again, we apply the simple approach of K-means clustering algorithm. We first apply K-means algorithm to have 22 clusters based on one million of 3D data points of $\left\{\left(X_{1},X_{2},X_{3}\right)\right\}$, $\left\{\left(X_{4},X_{5},X_{6}\right)\right\}$ and $\left\{\left(X_{7},X_{8},X_{9}\right)\right\}$, respectively. We specifically denote these three categorical variables as $X_{123}$, $X_{456}$ and $X_{789}$, respectively. Upon these three new covariate variables, we calculate CEs (of *Y*) under 1-feature and 2-feature settings, see Table 16. We consistently confirm that $X_{123}$ and $X_{456}$ are not conditionally dependent given *Y*. Therefore, the two feature triplets $\left(X_{1},X_{2},X_{3}\right)$ and $\left(X_{4},X_{5},X_{6}\right)$ are two separate chief and alternative collections of three order-1 major factors.

## 5. Conclusions

The most fundamental concept underlying all practical guidelines we have learned from the series of increasingly complex examples in this paper is: the comparability of evaluations of conditional entropy and mutual information critically rests on the equality of the dimensions of the contingency tables where these evaluations are carried out. Based on this comparability concept, the focal goal of the data analysis is then rephrased in terms of [C1: confirmable] criterion regrading presence and absence of major factors underlying a designated Re-Co dynamics. In other words, it is absolutely essential to note that there is no need for precise theoretical information measurements in real data analysis. Such [C1: confirmable] criterion pertaining to the discovery of major factor subsequently promotes all practical guidelines being centered around the task of confirming and debunking an existential collection of major factors of various orders. Since the presence and absence of such an existential collection of major factors indeed manifest the data’s authentic information content, from a data’s information content perspective, the task of data analysis as a whole is translated into the single issue of major factor selection.

Furthermore, all practical guidelines on evaluating mutual information, in particular, for our major factor selection protocol are largely recognized for ascertaining the [C1: confirmable] criterion against the effects of the curse of dimensionality or finite sample phenomenon. Practically, we learn to be sensitively aware of dangers of having low cell-counts in potentially occupied cells when evaluating entropy measures. We also develop clustering-based approaches to lessen the effect of the curse of dimensionality. After learning all these practical guidelines, we are confident in our applications of our major factor selection protocol and related Categorical Exploratory Data Analysis (CEDA) techniques on analyzing real-world structured data sets.

In many scientific fields, like biology, medicine, psychology and social sciences, many measurements are not always precisely metric. Even within a metric system, a continuous measurement is often grouped and converted into a discrete or even ordinal data format. That is, very fine-scale details of a data point is likely given up because it is either too costly to measure, or even can’t be measured, or needs to be discarded for practical computational considerations. Therefore, any structured data set is likely consisting of some features having incomparable measurement scales and some features having no scales at all. How to analyze such a data set in a coherent fashion is not at all a simple task. CEDA is a data analysis designed to be coherent with all features’ measurements. So, CEDA and its major factor selection protocol are developed to indeed embrace the ideal concept: Each single feature must allow to contribute its own authentic information locally, and then to congregate and weave patterns that reveal heterogeneity on global, median and fine scales levels.

To facilitate and carry out such a fundamental concept of data analysis, CEDA is exclusively resting on one simple fact: All data-types are embedded with the categorical nature. So all pieces of local information derived from all categorical or categorized features must be comparable. All these information pieces can be then woven together for the multiscale heterogeneity. By doing so, there are no man-made assumptions or structures needed in CEDA. So, information brought out by CEDA is authentic. That is, we can be free from the danger of generating misinformation via data analysis involving unrealistic assumptions or structures.

To achieve the aforementioned goals of CEDA via our major factor selection protocol, we definitely need stable and creditable evaluations of conditional entropy and mutual information underlying any targeted Re-Co dynamics of interest. That is why the practical guidelines learned in this paper become essential and significant. On the other hand, these practical guidelines also reveal aspects of flexibility and capability of CEDA and its major factor selection in helping scientists to extract intelligence from their own data sets.

As a final remark, we clearly demonstrate in this paper that, by reframing many key statistical topics in one Re-Co dynamics framework, CEDA and its major factor selection protocol not only can resolve the original data analysis tasks, but also, more importantly, can shed authentic lights on issues related to widely expanded frameworks containing the original statistical topics. This capability manifests the capability of CEDA and its major factor selection protocol for truly accommodating and resolving real-world scientific problems.

Finally, we conclude that the learned practical guidelines for evaluating CE and I[Re;Co] would allow scientists to effectively carry out CEDA and its major factor selection protocol to extract data’s visible and authentic information content, which is taken as the ultimate goal of data analysis.

## Figures and Tables

**Table 1:** Point estimations of mutual information $I[Y;V_{1}]$ with 0.1132 as its theoretical value: $I\left[Y;V_{1}\right]=H\left[Y\right]-H\left[Y|V_{1}\right]=1.5321-1.4189$, and null 95% confidence range (CR) of $I^{\left(K\right)}\left[Y;\varepsilon \right]$ with ε being the Binomial random variable under the null hypothesis.

N	Bin Size	H[Y]	$H[Y|V_{1}]$	$I[Y;V_{1}]$	95% CR of I[Y;ε]
2000	1 + 10 + 1	2.3993	2.2824	0.1168	[0.00254, 0.00298]
	1 + 20 + 1	3.0149	2.8951	0.1199	[0.00489, 0.00551]
	1 + 30 + 1	3.3782	3.2571	0.1211	[0.00757, 0.00836]
	1 + 100 + 1	4.4424	4.3043	0.1382	[0.02548, 0.02704]
	1 + 1000 + 1	6.2609	5.9149	0.3461	[0.26435, 0.26768]
20,000	1 + 10 + 1	2.4135	2.3011	0.1124	[0.00025, 0.00030]
	1 + 20 + 1	3.0350	2.9215	0.1135	[0.00050, 0.00057]
	1 + 30 + 1	3.3995	3.2856	0.1139	[0.00074, 0.00082]
	1 + 100 + 1	4.4807	4.3649	0.1157	[0.00243, 0.00258]
	1 + 1000 + 1	6.5310	6.3933	0.1377	[0.02591, 0.02637]

**Table 2:** Entropies of Example-2 calculated from contingency tables built based on K-means clustering compositions on the 2D data setting.

n	Bin Size	H[Y]	H[Y|X]	I[Y;X]	95% CR of I[Y;ε]
2000	12	2.3962	2.3866	0.0096	[0.00248, 0.00299]
	22	2.9722	2.9530	0.0192	[0.00487, 0.00544]
	32	3.3354	3.3123	0.0232	[0.00731, 0.00799]
	102	4.5430	4.4995	0.0434	[0.02576, 0.02711]
	1002	6.7989	6.4311	0.3678	[0.33761, 0.34149]
20,000	12	2.4208	2.4148	0.0060	[0.00024, 0.00029]
	22	2.9916	2.9816	0.0100	[0.00049, 0.00056]
	32	3.3500	3.3377	0.0123	[0.00074, 0.00081]
	102	4.5076	4.4899	0.0177	[0.00244, 0.00258]
	1002	6.8662	6.8236	0.0425	[0.02570, 0.02608]

**Table 3:** Entropies of Example-2 calculated from contingency tables built based on K-means clustering compositions on the 3D data setting.

n	Bin Size	H[Y]	H[Y|X]	I[Y;X]	95% CR of I[Y;ε]
2000	12	2.4411	2.4310	0.0101	[0.00260, 0.00303]
	22	3.0166	3.0028	0.0138	[0.00476, 0.00537]
	32	3.3706	3.3482	0.0224	[0.00732, 0.00812]
	102	4.5297	4.4771	0.0526	[0.02563, 0.02712]
	1002	6.8065	6.4558	0.3507	[0.33899, 0.34254]
20,000	12	2.4642	2.4620	0.0023	[0.00025, 0.00030]
	22	3.0425	3.0337	0.0088	[0.00047, 0.00053]
	32	3.4064	3.3958	0.0106	[0.00075, 0.00083]
	102	4.5307	4.5067	0.0241	[0.00246, 0.00258]
	1002	6.8551	6.7988	0.0563	[0.02582, 0.02632]

**Table 4:** Entropies of Example-2 calculated from contingency tables built based on K-means clustering compositions on the 4D data setting.

n	Bin Size	H[Y]	H[Y|X]	I[Y;X]	95% CR of I[Y;ε]
1000	12	2.4599	2.4556	0.0043	[0.00249, 0.00299]
	22	3.0612	3.0518	0.0094	[0.00477, 0.00536]
	32	3.4115	3.3911	0.0204	[0.00753, 0.00838]
	102	4.5065	4.4508	0.0557	[0.02565, 0.02717]
	1002	6.8162	6.4627	0.3535	[0.33696, 0.34110]
10,000	12	2.4756	2.4728	0.0029	[0.00026, 0.00032]
	22	3.0772	3.0736	0.0036	[0.00049, 0.00056]
	32	3.4456	3.4377	0.0079	[0.00073, 0.00081]
	102	4.5590	4.5347	0.0243	[0.00244, 0.00257]
	1002	6.8328	6.7697	0.0631	[0.02556, 0.02607]

**Table 5:** Entropies of two settings of Example-$2^{*}$ calculated from contingency tables built based on K-means clustering compositions with N= 20,000.

Data	Bin Size	H[Y]	H[Y|X]	I[Y;X]	95% CR of I[Y;ε]
1st mixture	12	2.4246	2.4233	0.0012	[0.00258, 0.00309]
	22	2.9958	2.9910	0.0048	[0.00506, 0.00575]
	32	3.3805	3.3725	0.0080	[0.00786, 0.00855]
	102	4.5481	4.5214	0.0267	[0.02622, 0.02747]
	1002	6.7953	6.4700	0.3252	[0.33811, 0.34153]
2nd mixture	12	2.4434	2.4375	0.0059	[0.00226, 0.00272]
	22	2.9943	2.9795	0.0147	[0.00529, 0.00602]
	32	3.3678	3.3518	0.0159	[0.00745, 0.00817]
	102	4.5485	4.5143	0.0342	[0.02542, 0.02690]
	1002	6.7975	6.4573	0.3403	[0.33702, 0.34059]

**Table 6:** (Y,X)∼MN((0,1),Σ) with ρ=0.0 and N=2000 (upper half), n= 20,000 (lower half).

Bin Size	Bin Size	H[Y]	H[Y|X]	I[Y;X]	95% CR of I[Y;ε]
Y = 12	X = 12	2.4135	2.3435	0.0700	[0.0637, 0.0669]
	X = 22	2.4135	2.2861	0.1273	[0.1231, 0.1274]
	X = 32	2.4135	2.2194	0.1940	[0.1863, 0.1916]
	X = 102	2.4135	1.7971	0.6164	[0.5714, 0.5787]
Y = 22	X = 12	3.0168	2.9014	0.1154	[0.1249, 0.1294]
	X = 22	3.0168	2.7650	0.2517	[0.2393, 0.2450]
	X = 32	3.0168	2.6319	0.3848	[0.3613, 0.3681]
	X = 102	3.0168	2.0360	0.9808	[0.9365, 0.9439]
Y = 32	X = 12	3.3910	3.1952	0.1958	[0.1899, 0.1951]
	X = 22	3.3910	3.0196	0.3714	[0.3587, 0.3656]
	X = 32	3.3910	2.8494	0.5416	[0.5143, 0.5209]
	X = 102	3.3910	2.1175	1.2736	[1.2040, 1.2106]
Y = 102	X = 12	4.5236	3.9131	0.6105	[0.5657, 0.5728]
	X = 22	4.5236	3.5339	0.9897	[0.9516, 0.9585]
	X = 32	4.5236	3.2717	1.2519	[1.2193, 1.2261]
	X = 102	4.5236	2.2962	2.2274	[2.1571, 2.1643]
Y = 12	X = 12	2.3392	2.3332	0.0059	[0.0060, 0.0063]
	X = 22	2.3392	2.3275	0.0116	[0.0115, 0.0119]
	X = 32	2.3392	2.3216	0.0175	[0.0172, 0.0177]
	X = 102	2.3392	2.2799	0.0592	[0.0578, 0.0588]
Y = 22	X = 12	2.9424	2.9311	0.0113	[0.0116, 0.0120]
	X = 22	2.9424	2.9215	0.0210	[0.0223, 0.0228]
	X = 32	2.9424	2.9109	0.0316	[0.0335, 0.0342]
	X = 102	2.9424	2.8334	0.1090	[0.1122, 0.1135]
Y = 32	X = 12	3.3311	3.3155	0.0157	[0.0174, 0.0179]
	X = 22	3.3311	3.2978	0.0333	[0.0334, 0.0341]
	X = 32	3.3311	3.2843	0.0468	[0.0496, 0.0505]
	X = 102	3.3311	3.1634	0.1677	[0.1675, 0.1690]
Y = 102	X = 12	4.5504	4.4933	0.0571	[0.0582, 0.0592]
	X = 22	4.5504	4.4401	0.1103	[0.1116, 0.1128]
	X = 32	4.5504	4.3836	0.1668	[0.1684, 0.1698]
	X = 102	4.5504	3.9991	0.5513	[0.5475, 0.5497]

**Table 7:** $\left(Y,X\right)∼MN\left(\tilde{0},\Sigma \right)$ with ρ=0.5 and *N* = 20,000.

Bin Size	Bin Size	H[Y]	H[Y|X]	I[Y;X]	95% CR of I[Y;Z]
Y = 12	X = 12	2.3317	2.1839	0.1478	[0.0058, 0.0062]
	X = 22	2.3317	2.1758	0.1559	[0.0114, 0.0119]
	X = 32	2.3317	2.1709	0.1609	[0.0175, 0.0180]
	X = 102	2.3317	2.1270	0.2048	[0.0578, 0.0588]
Y = 22	X = 12	2.9543	2.7995	0.1548	[0.0116, 0.0120]
	X = 22	2.9543	2.7852	0.1692	[0.0224, 0.0230]
	X = 32	2.9543	2.7750	0.1793	[0.0336, 0.0344]
	X = 102	2.9543	2.7018	0.2525	[0.1125, 0.1139]
Y = 32	X = 12	3.3654	3.2043	0.1611	[0.0172, 0.0178]
	X = 22	3.3654	3.1864	0.1790	[0.0332, 0.0339]
	X = 32	3.3654	3.1708	0.1945	[0.0492, 0.0501]
	X = 102	3.3654	3.0555	0.3099	[0.1672, 0.1688]
Y = 102	X = 12	4.5415	4.3416	0.1999	[0.0583, 0.0590]
	X = 22	4.5415	4.2849	0.2565	[0.1117, 0.1131]
	X = 32	4.5415	4.2344	0.3070	[0.1654, 0.1668]
	X = 102	4.5415	3.8806	0.6609	[0.5488, 0.5513]

**Table 8:** Evaluations of entropy, conditional entropy and mutual information under the half-sine simulation study.

Bin Size	Bin Size	H[Y]	H[Y|X]	I[Y;X]	95% CR of I[Y;ε]
Y = 12	X = 12	2.4840	1.7450	0.7391	[0.00600, 0.00632]
	X = 22	2.4840	1.7326	0.7514	[0.01137, 0.01183]
	X = 32	2.4840	1.7237	0.7603	[0.01690, 0.01742]
	X = 102	2.4840	1.6977	0.7863	[0.05704, 0.05804]
Y = 22	X = 12	3.0881	2.3131	0.7749	[0.01151, 0.01189]
	X = 22	3.0881	2.2975	0.7906	[0.02205, 0.02264]
	X = 32	3.0881	2.2853	0.8028	[0.03305, 0.03387]
	X = 102	3.0881	2.2369	0.8512	[0.11254, 0.11387]
Y = 32	X = 12	3.4499	2.6679	0.7819	[0.01689, 0.01743]
	X = 22	3.4499	2.6466	0.8033	[0.03272, 0.03343]
	X = 32	3.4499	2.6335	0.8163	[0.04928, 0.05009]
	X = 102	3.4499	2.5559	0.8940	[0.17143, 0.17303]
Y = 102	X = 12	4.6133	3.7972	0.8161	[0.05663, 0.05757]
	X = 22	4.6133	3.7550	0.8583	[0.11237, 0.11375]
	X = 32	4.6133	3.7194	0.8939	[0.17072, 0.17235]
	X = 102	4.6133	3.5164	1.0969	[0.56831, 0.57063]

**Table 9:** Evaluations of entropy, conditional entropy and mutual information under the whole-sine simulation study.

Bin Size	Bin Size	H[Y]	H[Y|X]	I[Y;X]	95% CR of I[Y;ε]
Y = 12	X = 12	2.4807	2.1916	0.2890	[0.0061, 0.0064]
	X = 22	2.4807	2.1822	0.2984	[0.0115, 0.0120]
	X = 32	2.4807	2.1757	0.3050	[0.0171, 0.0176]
	X = 102	2.4807	2.1310	0.3497	[0.0567, 0.0577]
Y = 22	X = 12	3.0692	2.7651	0.3042	[0.0114, 0.0118]
	X = 22	3.0692	2.7517	0.3175	[0.0223, 0.0229]
	X = 32	3.0692	2.7426	0.3266	[0.0333, 0.0341]
	X = 102	3.0692	2.6671	0.4022	[0.1133, 0.1147]
Y = 32	X = 12	3.4398	3.1293	0.3105	[0.0170, 0.0175]
	X = 22	3.4398	3.1094	0.3303	[0.0331, 0.0338]
	X = 32	3.4398	3.0980	0.3417	[0.0493, 0.0502]
	X = 102	3.4398	2.9917	0.4481	[0.1717, 0.1735]
Y = 102	X = 12	4.5698	4.2185	0.3513	[0.0577, 0.0587]
	X = 22	4.5698	4.1752	0.3946	[0.1118, 0.1130]
	X = 32	4.5698	4.1233	0.4466	[0.1679, 0.1694]
	X = 102	4.5698	3.7851	0.7848	[0.5577, 0.5602]

**Table 10:** Experiment with $Y=X_{1}+\sin\left(2\pi \left(X_{2}+X_{3}\right)\right)+N\left(0,1\right)/10$ and *N* = 10,000. Each categorized 1-features has 12 bins, so a *k*-feature has ${\left(12\right)}^{k}$*k*D hypercubes.

1-Feature	CE	SCE-Drop	2-Feature	CE	SCE-Drop	3-Feature	CE	SCE-Drop	4-Feature	CE	SCE-Drop
X1	2.2315	0.2322	X1_X2	2.1671	0.0644	X1_X2_X3	0.8362	0.8431	X1_X2_X3_X4	0.1762	0.6599
X2	2.4579	0.0057	X1_X3	2.1647	0.0667	X1_X2_X4	1.4451	0.7219			
X3	2.4575	0.0062	X1_X4	2.1685	0.0630	X1_X3_X4	1.4531	0.7115			
X4	2.4557	0.0079	X2_X3	1.6793	0.7781	X2_X3_X4	1.2263	0.4530			
			X2_X4	2.3780	0.0777						
			X3_X4	2.3831	0.0726						

**Table 11:** Experiment with $Y=X_{1}+\sin\left(2\pi \left(X_{2}+X_{3}+X_{4}\right)\right)+N\left(0,1\right)/10$ and *N* = 10,000. Each categorized 1-features has 12 bins, so a *k*-feature has ${\left(12\right)}^{k}$*k*D hypercubes.

1-Feature	CE	CE-Drop	2-Feature	CE	SCE-Drop	3-Feature	CE	SCE-Drop	4-Feature	CE	SCE-Drop
X1	2.2299	0.2295	X1_X2	2.1636	0.0662	X1_X2_X3	1.4444	0.7191	X1_X2_X3_X4	0.1945	1.0367
X2	2.4539	0.0055	X1_X3	2.1671	0.0627	X1_X2_X4	1.4576	0.7059			
X3	2.4550	0.0044	X1_X4	2.1645	0.0653	X1_X3_X4	1.4473	0.7171			
X4	2.4529	0.0065	X2_X3	2,3800	0.0739	X2_X3_X4	1.2313	1.1455			
			X2_X4	2.3800	0.0728						
			X3_X4	2.3768	0.0760						

**Table 12:** Experiment with $Y=X_{1}+\sin\left(2\pi \left(X_{2}+X_{3}+X_{4}\right)\right)+N\left(0,1\right)/10$ and N=1000. Each categorized 1-features has 12 bins, so a *k*-feature has ${\left(12\right)}^{k}$*k*D hypercubes.

1-Feature	CE	CE-Drop	2-Feature	CE	SCE-Drop	3-Feature	CE	SCE-Drop	4-Feature	CE	SCE-Drop
X1	2.1873	0.2572	X1_X2	1.5863	0.6010	X1_X2_X3	0.3657	1.2022	X1_X2_X3_X4	0.0207	0.2947
X2	2.3945	0.0500	X1_X3	1.5679	0.6193	X1_X2_X4	0.3155	1.2601			
X3	2.3789	0.0655	X1_X4	1.5757	0.6116	X1_X3_X4	0.3258	1.2421			
X4	2.3819	0.0625	X2_X3	1.6502	0.7286	X2_X3_X4	0.3553	1.2718			
			X2_X4	1.6272	0.7547						
			X3_X4	1.6387	0.7402						

**Table 13:** Exploring the presence of $X_{234}$ as an order-3 major factor of $Y=X_{1}+\sin\left(2\pi \left(X_{2}+X_{3}+X_{4}\right)\right)+N\left(0,1\right)/10$ with N=1000 with respect to 2 and 4 choices of numbers of clusters of *Y* and $X_{234}$, respectively. The confidence intervals are calculated based on 100 simulations.

*Y*’s Size	$X_{234}$’s Size	$H[Y|X_{234}]$	Mean of $H[Y|X_{234}^{\varepsilon }]$	95% CR of $H[Y|X_{234}^{\varepsilon }]$
12	12	2.345	2.394	[2.393, 2.396]
	36	2.039	2.195	[2.192, 2.198]
	72	1.783	1.981	[1.978, 1.984]
	144	1.409	1.652	[1.648, 1.655]
32	12	3.141	3.192	[3.190, 3.194]
	36	2.651	2.790	[2.787, 2.794]
	72	2.180	2.385	[2.382, 2.388]
	144	1.720	1.888	[1.885, 1.892]

**Table 14:** Example-6 with $N=10^{6}$. Each categorized 1-features has 22 bins, so a *k*-feature has ${\left(22\right)}^{k}$*k*D hypercubes.

1-Feature	CE	2-Feature	CE	3-Feature	CE	4-Feature	CE	5-Feature	CE	6-Feature	CE
X6	2.3351	X4_X6	2.1321	X1_X2_X3	0.7543	X1_X2_X3_X7	0.5602	X1_X2_X3_X7_X8	0.1020	X1_X2_X3_X7_X8_X9	0.0065
X3	2.7295	X1_X6	2.2439	X4_X5_X6	1.0746	X1_X2_X3_X6	0.6201	X1_X2_X3_X6_X9	0.1723	X1_X2_X3_X6_X7_X8	0.0132
X1	2.7308	X1_X2	2.3184	X1_X2_X6	2.0049	X4_X5_X6_X8	0.8789	X1_X7_X8_X9_X10	0.2255	X1_X4_X5_X7_X8_X9	0.0150
X2	2.7310	X6_X7	2.3309	X1_X4_X6	2.0239	X1_X4_X5_X6	0.8965	X4_X5_X6_X8_X9	0.2355	X1_X2_X3_X5_X6_X8	0.0202
X9	2.9879	X3_X7	2.7010	X4_X6_X7	2.0771	X2_X3_X5_X7	1.4054	X1_X4_X5_X6_X8	0.2681	X2_X3_X6_X7_X8_X9	0.0211
X8	2.9880	X3_X4	2.7012	X3_X6_X9	2.1765	X4_X6_X7_X9	1.4468	X5_X6_X7_X8_X9	0.2719	X4_X5_X6_X7_X8_X9	0.0240
X7	2.9882	X7_X8	2.9516	X1_X2_X7	2.2328	X6_X7_X8_X9	1.4605	X2_X5_X6_X8_X9	0.3022	X1_X4_X5_X6_X7_X8	0.0280
X4	2.9882	X5_X7	2.9520	X6_X7_X8	2.2572	X1_X6_X8_X9	1.4752	X1_X4_X6_X7_X8	0.3035	X1_X2_X5_X6_X8_X9	0.0280
X5	2.9883	X4_X5	2.9522	X1_X7_X9	2.5849	X1_X7_X8_X9	1.5458	X1_X2_X4_X5_X6	0.3236	X1_X2_X4_X5_X6_X8	0.0329
X10	2.9883	X4_X7	2.9523	X7_X8_X9	2.8139	X7_X8_X9_X10	1.6278	X1_X2_X5_X6_X9	0.3427	X1_X2_X3_X4_X5_X6	0.0584

**Table 15:** Results of parameters in linear regression with categorized data.

	Estimate	Std. Error	t Value	Pr(>|t|)
(intercept)	−0.776	0.013	−59.57	0.000
X1	0.334	0.004	819.68	0.000
X2	0.334	0.004	820.21	0.000
X3	0.334	0.004	820.05	0.000
X4	−0.232	0.004	−568.08	0.000
X5	−0.231	0.004	−566.27	0.000
X6	0.528	0.008	624.12	0.000
X7	−0.0002	0.001	−0.94	0.3462
X8	0.0001	0.001	0.56	0.5735
X9	−0.0002	0.001	−1.40	0.1622
X10	−0.0002	0.001	−1.13	0.2567

**Table 16:** Escaping from the curse of dimensionality in Example-6.

Experiments	1-Feature	CE	2-Feature	CE
L0.2	$X_{123}$	1.9317	$X_{123}$_$X_{456}$	1.8206
	$X_{456}$	2.4734	$X_{123}$_$X_{789}$	1.9195
	$X_{789}$	2.9450	$X_{456}$_$X_{789}$	2.4555

## Data Availability

The data presented in this study are available on request from the corresponding author.

## References

[1] Faes L., Porta A., Wibral M., Vicente R., Lizier J. (2014). Conditional Entropy-Based Evaluation of Information Dynamics in Physiological Systems. Directed Information Measures in Neuroscience.

[2] Wibral M., Vicente R., Lizier J. (2014). Directed Information Measures in Neuroscience.

[3] Child D. (2006). The Essentials of Factor Analysis.

[4] Contreras-Reyes J.E., Hernandez-Santoro C. (2020). Assessing Granger-Causality in the Southern Humboldt Current Ecosystem Using Cross-Spectral Methods. Entropy.

[5] Gell-Mann M. (1995). What is complexity?. Complexity.

[6] Adami C. (2002). What is Complexity?. BioEssays.

[7] Anderson P.W. (1972). More is different. Science.

[8] Lehmann E.L., Romano J.P. (2005). Testing Statistical Hypotheses.

[9] Fisher R.A. (1925). Statistical Methods for Research Workers.

[10] Scheffé H. (1959). The Analysis of Variance.

[11] McCullagh P., Nelder J. (1989). Generalized Linear Models.

[12] Christensen R. (1997). Log-Linear Models and Logistic Regression.

[13] Fushing H., Chou E.P. (2021). Categorical Exploratory Data Analysis: From Multiclass Classification and Response Manifold Analytics perspectives of baseball pitching dynamics. Entropy.

[14] Fushing H., Chou E.P., Chen T.-L. (2021). Mimicking complexity of structured data matrix’s information content: Categorical Exploratory Data Analysis. Entropy.

[15] Chen T.-L., Chou E.P., Fushing H. (2022). Categorical Nature of Major Factor Selection via Information Theoretic Measurements. Entropy.

[16] Chou E.P., Chen T.-L., Fushing H. (2022). Unraveling Hidden Major Factors by Breaking Heterogeneity into Homogeneous Parts within Many-System Problems. Entropy.

[17] Fushing H., Chou E.P., Chen T.-L. (2022). Multiscale major factor selections for complex system data with structural dependency and heterogeneity. arXiv.

[18] Cover T.M., Thomas J.A. (1991). Elements of Information Theory.

[19] Paninski L. (2003). Estimation of Entropy and Mutual Information. Neural Comput..

[20] Kraskov A., Stögbauer H., Grassberger P. (2004). Estimating mutual information. Phys. Rev. E.

[21] Brown G., Pocock A., Zhao M., Lujan M. (2012). Conditional likelihood maximisation: A unifying framework for information theoretic feature selection. J. Mach. Learn. Res..

[22] Vergara J., Estevez P. (2014). A review of feature selection methods based on mutual information. Neural Comput. Appl..

[23] Bennasar M., Hicks Y., Setchi R. (2015). Feature selection using Joint Mutual Information Maximisation. Expert Syst. Appl..

[24] Zhao X., Shang P., Huang J. (2017). Mutual-information matrix analysis for nonlinear interactions of multivariate time series. Nonlinear Dyn..

[25] Fushing H., Roy T. (2018). Complexity of Possibly-gapped Histogram and Analysis of Histogram (ANOHT). R. Soc. Open Sci..

[26] Grenander U. (1981). Abstract Inference.

